# Associations between corneal curvature and other anterior segment biometrics in young myopic adults

**DOI:** 10.1038/s41598-024-59037-z

**Published:** 2024-04-09

**Authors:** Li Jiang, Zijing Du, Wei Sun, Shanqing Zhu, Lu Xiong, Xuejun Fang, Jin Zhou, Qingsong Zhang, Xiaohua Lei, Qingyan Zeng, Zheng Wang, Yijun Hu

**Affiliations:** 1 Aier Eye Hospital of Wuhan University (Wuhan Aier Eye Hospital), Wuhan, China; 2 Refractive Surgery Center, Hankou Aier Eye Hospital, Wuhan, China; 3 Guangdong Eye Institute, Department of Ophthalmology, Guangdong Provincial People’s Hospital (Guangdong Academy of Medical Sciences), Southern Medical University, Guangzhou, China; 4 Aier Institute of Refractive Surgery, Refractive Surgery Center, Guangzhou Aier Eye Hospital, Guangzhou, China; 5 Refractive Surgery Center, Shenyang Aier Eye Hospital, Shenyang, China; 6 Refractive Surgery Center, Chengdu Aier Eye Hospital, Chengdu, China

**Keywords:** Ocular biometrics, Anterior segment biometrics, Corneal biometrics, Corneal curvature (CC), Myopia, Refractive surgery, Medical research, Eye diseases, Public health

## Abstract

To investigate the associations between corneal curvature (CC) and other anterior segment biometrics in young myopic adults. In this retrospective multi-center study, 7893 young myopic adults were included. CC and other anterior segment biometrics were measured by Scheimpflug imaging (Pentacam). CC was defined as SimK at central 3 mm area, and other anterior segment biometrics included white-to-white corneal diameter (WTW), central corneal thickness (CCT), corneal volume (CV) at 3 mm, 5 mm, and 7 mm area, anterior corneal astigmatism (ACA), posterior corneal astigmatism (PCA), anterior corneal eccentricity (ACE) and asphericity (ACAP), posterior corneal eccentricity (PCE) and asphericity (PCAP), anterior chamber depth (ACD), and anterior chamber volume (ACV). Univariate regression analyses were used to assess the associations between CC and other anterior segment biometrics, and multivariate regression analyses were further performed to adjusted for age, gender and spherical equivalent. CC was higher in patients of female gender and higher myopia (all *P* < 0.05). Eyes in higher CC quartiles had lower WTW, thinner CCT, lower CV at 3 mm and 5 mm, lower ACD, and lower ACV (all *P* < 0.001), but had larger ACA, larger PCA, less PCE and less PCAP (all *P* < 0.001), compared to eyes in lower CC quartiles. The trends of CV at 7 mm, ACE and ACAP were inconsistent in different CC quartiles. After adjusting for age, gender and spherical equivalent with multivariate linear regression, CC was positively correlated to CV at 7 mm (β_s_ = 0.069), ACA (β_s_ = 0.194), PCA (β_s_ = 0.187), ACE (β_s_ = 0.072), PCAP (β_s_ = 0.087), and ACD (β_s_ = 0.027) (all *P* < 0.05), but was negatively correlated to WTW (β_s_ = − 0.432), CCT (β_s_ = − 0.087), CV-3 mm (β_s_ = − 0.066), ACAP (β_s_ = − 0.043), PCE (β_s_ = − 0.062), and ACV (β_s_ = − 0.188) (all *P* < 0.05). CC was associated with most of the other anterior segment biometrics in young myopic adults. These associations are important for better understanding of the interactions between different anterior segment structures in young myopic patients, and are also useful for the exploration of the pathogenesis of myopia.

## Introduction

Myopia is a common ocular health issue with an estimated global prevalence of 28.3%, especially affecting children and young adults in east and south-east Asian countries^1,2^. During the development of myopia, ocular biometrics are believed to play crucial roles^3,4^. Although the key mechanism of myopia mainly involves elongation of the eyeball and vitreous cavity^5^, changes of anterior segment structures are also suggested to play roles in myopia^6,7^. Moreover, different ocular biometrics are often inter-correlated in myopia, suggesting interplay of different ocular structures in myopia development^8,9^.

The cornea is the foremost refractive tissue of the eye. It provides about two third of the refractive power of the eye. Corneal refractive power can be measured as corneal curvature (CC) which is commonly shown as simulated keratometry (SimK). In general and aged populations, it was shown that corneal refractive power was correlated to axial length (AL)^10,11^ and some of the anterior segment biometrics, such as central corneal thickness (CCT) and anterior chamber depth (ACD)^10^.

In patients with myopia, CC was also correlated to AL^12,13^. However, the correlations of CC and other anterior segment biometrics in myopia were rarely reported^14^. In the present study with data from multiple centers, we have found correlations between CC and other anterior segment biometrics in young myopic adults. The findings may reveal the relationships of CC and other anterior segment biometrics in myopia.

## Methods

### Participants

The participants were described in previous studies^8,15,16^, which were approved by the Institutional Review Board (IRB) of Guangzhou Aier Eye Hospital (GZ), Shenyang Aier Eye Hospital (SY), Wuhan Aier Eye Hospital (WH), Chengdu Aier Eye Hospital (CD) and Hankou Aier Eye Hospital (HK). The study were conducted according to the tenets of the Declaration of Helsinki, without the needs for informed consent, because no participants could be identified from the data^8,15,16^. We included right eye data of young myopic adults aged 18–40 and with a spherical equivalent (SE) ≤ -0.50 diopter (D) and good quality Pentacam scans. Patients were excluded if they had coexisting corneal diseases, such as keratoconus (a significantly asymmetrical bowtie on the curvature map, a posterior elevation value of ≥  + 15 at the thinnest point with red spot on Belin/Ambrosio Enhanced Ectasia Display)^8,17^ and forme fruste keratoconus (such as the follow eye of patients with unilateral keratoconus)^8,18^, previous ocular surgery or trauma, severe dry eye, uveitis, glaucoma, wearing soft contact lenses within 2 weeks or rigid gas-permeable lenses within 1 month before examination^8,15,16^.

### Examinations

All patients underwent a set of ocular examinations, including best-corrected visual acuity (BCVA), intraocular pressure (IOP), manifest and cycloplegic refraction, slit-lamp examination of the anterior segment, corneal topography, and Pentacam measurements^8,15,16^. Myopia was divided into three groups according to the SE: low myopia (− 3.00 D < SE ≤  − 0.50 D), moderate myopia (− 6.00 D < SE ≤  − 3.00 D) and high myopia (SE ≤  − 6.00 D).

Pentacam (Oculus GmbH, Wetzlar, Germany) was used to obtain CC (SimK at central 3 mm area) and other anterior segment biometrics, including white-to-white corneal diameter (WTW), CCT, corneal volume (CV) at 3 mm, 5 mm, and 7 mm area, anterior corneal astigmatism (ACA), posterior corneal astigmatism (PCA), anterior corneal eccentricity (ACE) and asphericity (ACAP), posterior corneal eccentricity (PCE) and asphericity (PCAP), anterior chamber depth (ACD), and anterior chamber volume (ACV). All of the CC and anterior segment parameters were recorded before mydriasis.

### Statistical analysis

Normal distributions of the data were tested by Kolmogorov–Smirnov (KS) test. Basic characteristics of different CC quartiles, CC in different age and myopia groups, and other anterior segment biometrics in different CC quartiles were compared by Kruskal–Wallis test. Multivariate linear regression with the standardized regression coefficients (β_s_) were used to test the correlations of CC and other ocular biometrics adjusted for age, gender and SE. *P* < 0.05 was considered to be statistically significant.

### Ethics statement

This study was conformed to the tenets of the Declaration of Helsinki and was approved by the Institutional Review Board (IRB) of Guangzhou Aier Eye Hospital (GZ), Shenyang Aier Eye Hospital (SY), Wuhan Aier Eye Hospital (WH), Chengdu Aier Eye Hospital (CD) and Hankou Aier Eye Hospital (HK). This study was only a review of medical records from which patients could not be identified, so the IRBs decided to waive the requirement to get informed consent.

## Results

### Basic characteristics

**Figure 1:**
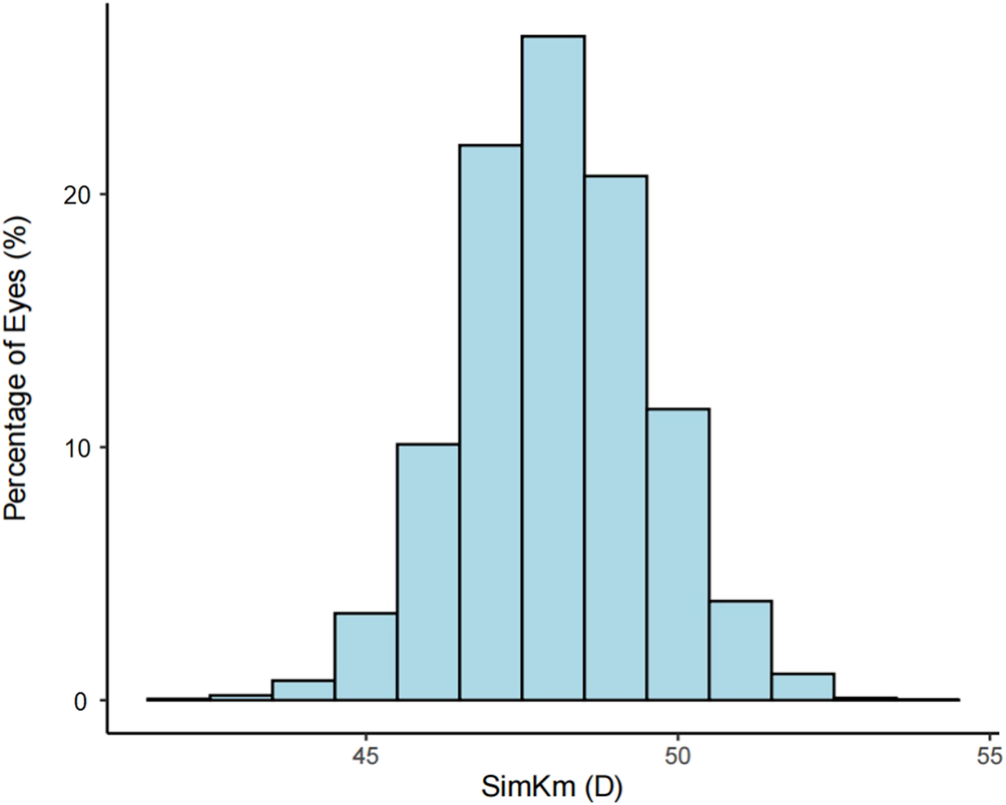
Frequency distribution of CC. *CC* corneal curvature, *D* diopter.

CC of the participants was normally distributed (Fig. 1). Age, sex, spherical error, astigmatism, and SE in different CC quartiles were significantly different (Table 1). Older age and more females were observed in higher CC quartiles (*P* < 0.001). More spherical error, astigmatism, and SE were also present in higher CC quartiles (*P* < 0.001).

**Table 1:** Demography of the eyes in different CC quartiles.

Number of eyes	1st quartile	2nd quartile	3rd quartile	4th quartile	P value
	1975	1973	1972	1973	
CC (D)*	41.42 ± 0.68	42.66 ± 0.25	43.55 ± 0.27	44.83 ± 0.66	< 0.001
Age (years)*	23.98 ± 5.04	24.69 ± 5.24	25.43 ± 5.42	26.45 ± 5.60	< 0.001
Female (no, %)	544 (15.65%)	758 (21.80%)	998 (28.70%)	1177 (33.85%)	< 0.001
Spherical error (D)*	− 4.52 ± 1.80	− 4.63 ± 1.83	− 4.87 ± 1.97	− 5.09 ± 2.24	< 0.001
Astigmatism (D)*	− 0.66 ± 0.53	− 0.74 ± 0.61	− 0.79 ± 0.65	− 0.81 ± 0.66	< 0.001
Spherical equivalent (D)*	− 4.82 ± 1.85	− 4.97 ± 1.90	− 5.24 ± 2.05	− 5.48 ± 2.32	< 0.001

*CC* corneal curvature, *D* diopter.

*Presented as mean ± standard deviation.

### CC in different sex, age and myopia groups

CC was significantly different in different age, sex and myopia groups (Tables 2 and 3). CC was higher in females than in males in every age group and myopia group (*P* < 0.001). In every age group and sex group, CC was higher in eyes with high myopia, compared to those with mild or moderate myopia (*P* < 0.05).

**Table 2:** Distribution of corneal curvature in different myopia groups*.

	Mild myopia (n = 945)	Moderate myopia (n = 4524)	High myopia (n = 2424)	P value
Age (years)				
Age 18–22, (n = 3170)	42.75 ± 1.34	42.76 ± 1.27	43.07 ± 1.34	< 0.001
Age 23–27, (n = 2284)	42.95 ± 1.40	43.04 ± 1.33	43.33 ± 1.30	< 0.001
Age 28–33, (n = 1750)	43.21 ± 1.29	43.36 ± 1.32	43.56 ± 1.23	0.001
Age 34–40, (n = 689)	43.12 ± 1.31	43.56 ± 1.39	43.50 ± 1.38	0.026
P value	< 0.001	< 0.001	< 0.001	
Sex				
Female (n = 3477, 44.05%)	43.39 ± 1.27	43.46 ± 1.29	43.60 ± 1.28	0.001
Male (n = 4416, 55.95%)	42.75 ± 1.34	42.75 ± 1.30	42.97 ± 1.30	< 0.001
P value	< 0.001	< 0.001	< 0.001	

*Presented as mean ± standard deviation in diopter.

**Table 3:** Distribution of corneal curvature in different age and gender groups*.

	Female (n = 3477)	Male (n = 4416)	P value
Age (years)			
Age 18–22, (n = 3170)	43.31 ± 1.24	42.71 ± 1.29	< 0.001
Age 23–27, (n = 2284)	43.45 ± 1.29	42.81 ± 1.30	< 0.001
Age 28–33, (n = 1750)	43.59 ± 1.27	43.04 ± 1.31	< 0.001
Age 34–40, (n = 689)	43.76 ± 1.33	43.10 ± 1.36	< 0.001
P value	< 0.001	< 0.001	

*Presented as mean ± standard deviation in diopter.

### Other anterior segment biometrics in different CC quartiles

**Figure 2:**
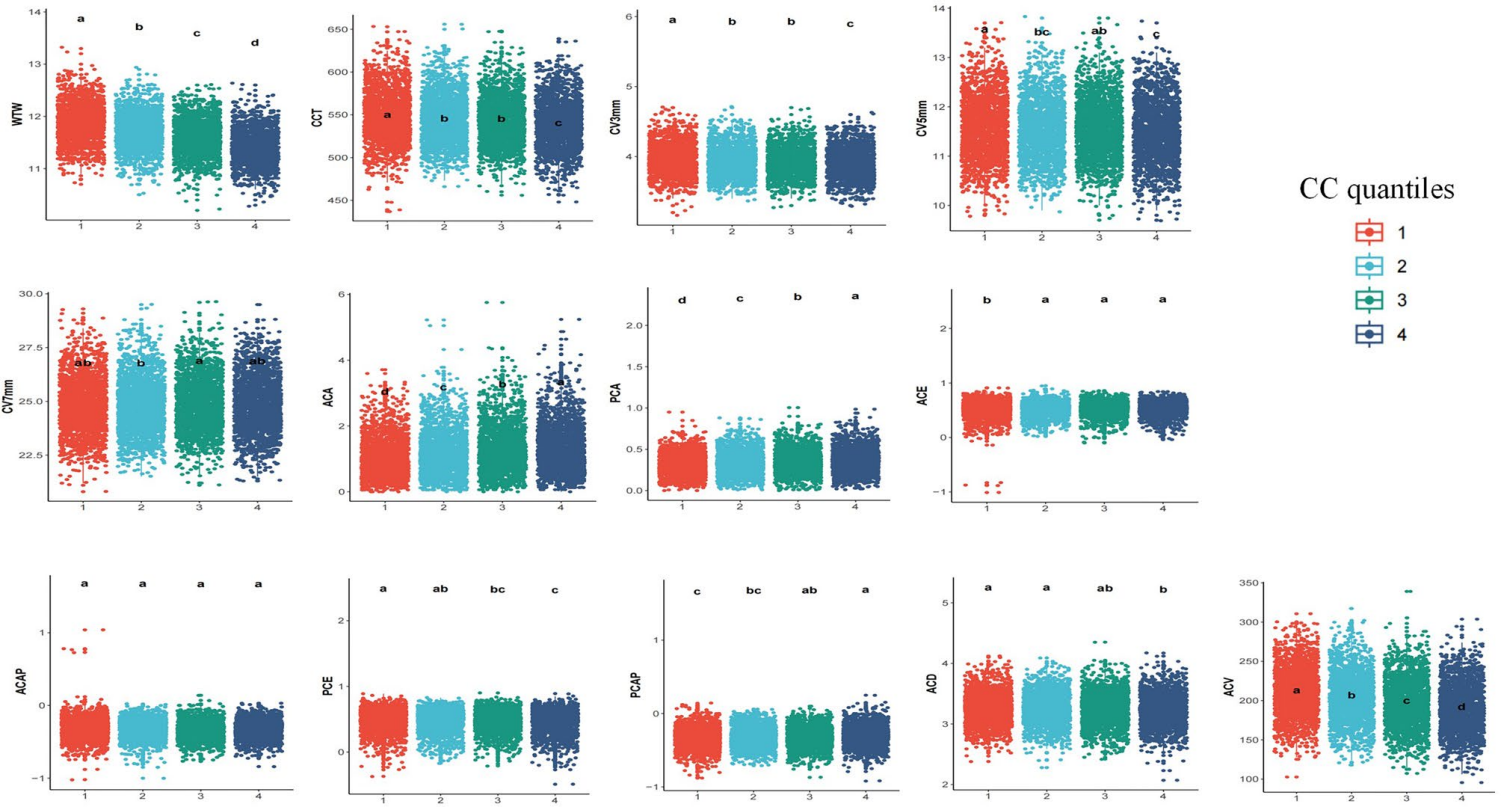
Multiple comparisons of Other anterior segment biometrics in different CC quartiles. Multiple comparisons were conducted using the Dunn-Bonferroni test. Using letters (e.g. a, ab, b) to represent different levels of significance among groups. The meaning of these letters is as follows: If two groups share the same letter (e.g. a and a), there is no significant difference between them. If two groups have different letters (e.g. a and b), there is a significant difference between them. If two groups share the same letter, but there are other groups with different letters (e.g. ab and a), it means that groups with the same letter do not have significant differences between them, however, there are significant differences between them and groups with different letters. *CC* corneal curvature, *SD* standard deviation, *WTW* white-to-white corneal diameter, *CCT* central corneal thickness, *CV* corneal volume, *ACA* anterior corneal astigmatism, PCA posterior corneal astigmatism, ACE anterior corneal eccentricity, *ACAP* anterior corneal asphericity, *PCE* posterior corneal eccentricity, *PCAP* posterior corneal asphericity, *ACD* anterior chamber depth, *ACV* anterior chamber volume.

Other anterior segment biometrics were statistically different in eyes of different CC quartiles (Table 4 and Fig. 2). Eyes in higher CC quartile had smaller WTW, thinner CCT, smaller CV-3 mm and CV-5 mm, higher ACA and PCA, lower PCE, higher PCAP, smaller ACD and ACV (*P* < 0.001). Although there were significant differences in CV-7 mm and ACE in different CC quartiles (*P* < 0.05), the trends were inconsistent.

**Table 4:** Anterior segment biometrics of the eyes in different CC quartile.

Number of eyes	1st quartile	2nd quartile	3rd quartile	4th quartile	P value
	1975	1973	1972	1973	
Corneal diameter, thickness and volume (mean ± SD)					
WTW (mm)	11.88 ± 0.36	11.72 ± 0.34	11.60 ± 0.33	11.42 ± 0.33	< 0.001
CCT (μm)	548.34 ± 29.07	543.88 ± 28.05	543.45 ± 27.43	538.78 ± 28.84	< 0.001
CV-3 mm (mm^3^)	3.96 ± 0.21	3.93 ± 0.21	3.93 ± 0.20	3.90 ± 0.21	< 0.001
CV-5 mm (mm^3^)	11.58 ± 0.60	11.52 ± 0.59	11.54 ± 0.58	11.48 ± 0.61	< 0.001
CV-7 mm (mm^3^)	24.80 ± 1.27	24.78 ± 1.25	24.90 ± 1.24	24.88 ± 1.29	0.003
Corneal astigmatism, eccentricity and asphericity (mean ± SD)					
ACA (D)	0.95 ± 0.53	1.06 ± 0.60	1.15 ± 0.63	1.20 ± 0.65	< 0.001
PCA (D)	0.31 ± 0.12	0.33 ± 0.13	0.35 ± 0.14	0.36 ± 0.14	< 0.001
ACE	0.52 ± 0.16	0.54 ± 0.12	0.54 ± 0.12	0.54 ± 0.12	0.009
ACAP	− 0.32 ± 0.14	− 0.32 ± 0.12	− 0.33 ± 0.12	− 0.32 ± 0.12	0.10
PCE	0.50 ± 0.16	0.49 ± 0.16	0.48 ± 0.16	0.47 ± 0.17	< 0.001
PCAP	− 0.33 ± 0.14	− 0.32 ± 0.14	− 0.31 ± 0.13	− 0.31 ± 0.14	< 0.001
Anterior chamber depth and volume (mean ± SD)					
ACD (mm)	3.26 ± 0.25	3.26 ± 0.26	3.24 ± 0.26	3.22 ± 0.26	< 0.001
ACV (mm^3^)	211.29 ± 31.14	205.09 ± 30.60	198.28 ± 30.38	190.49 ± 30.70	< 0.001

*CC* corneal curvature, *SD* standard deviation, *WTW* white-to-white corneal diameter, *CCT* central corneal thickness, *CV* corneal volume, *ACA* anterior corneal astigmatism, *PCA* posterior corneal astigmatism, *ACE* anterior corneal eccentricity, *ACAP* anterior corneal asphericity, *PCE* posterior corneal eccentricity, *PCAP* posterior corneal asphericity, *ACD* anterior chamber depth, *ACV* anterior chamber volume.

### Correlations of CC and other anterior segment biometrics

In univariate linear regression analyses, CC was significantly correlated to all of the other anterior segment biometrics (all *P* < 0.001). In multivariate linear regression analyses adjusting for age, gender and SE, CC was positively correlated to CV-7 mm (β_s_ = 0.069), ACA (β_s_ = 0.194), PCA (β_s_ = 0.187), ACE (β_s_ = 0.072), PCAP (β_s_ = 0.087), and ACD (β_s_ = 0.027) (all *P* < 0.05), but was negatively correlated to WTW (β_s_ = − 0.432), CCT (β_s_ = − 0.087), CV-3 mm (β_s_ = − 0.066), ACAP (β_s_ = − 0.043), PCE (β_s_ = − 0.062), and ACV (β_s_ = − 0.188) (all *P* < 0.05) (Table 5).

**Table 5:** Correlations of the anterior segment biometrics with CC.

	Univariate linear regression with CC		Multivariate linear regression with CC adjusted for age, gender and SE	
	Standard beta	P value*	Standard beta	P value*
Corneal diameter, thickness and volume				
WTW (mm)	− 0.475	< 0.001	− 0.432	< 0.001
CCT (μm)	− 0.114	< 0.001	− 0.087	< 0.001
CV-3 mm (mm^3^)	− 0.093	< 0.001	− 0.066	< 0.001
CV-5 mm (mm^3^)	− 0.044	< 0.001	− 0.016	0.161
CV-7 mm (mm^3^)	0.044	< 0.001	0.069	< 0.001
Corneal astigmatism, eccentricity and asphericity				
ACA (D)	0.165	< 0.001	0.194	< 0.001
PCA (D)	0.151	< 0.001	0.187	< 0.001
ACE	0.070	< 0.001	0.072	< 0.001
ACAP	− 0.038	< 0.001	− 0.043	< 0.001
PCE	− 0.054	< 0.001	− 0.062	< 0.001
PCAP	0.075	< 0.001	0.087	< 0.001
Anterior chamber depth and volume				
ACD (mm)	− 0.06	< 0.001	0.027	0.013
ACV (mm^3^)	− 0.261	< 0.001	− 0.188	< 0.001

*CC* corneal curvature, *WTW* white-to-white corneal diameter, *SE* spherical equivalent, *CI* confidential interval, *CCT* central corneal thickness, *CV* corneal volume, *ACA* anterior corneal astigmatism, *PCA* posterior corneal astigmatism, *ACE* anterior corneal eccentricity, *ACAP* anterior corneal asphericity, *PCE* posterior corneal eccentricity, *PCAP* posterior corneal asphericity, *ACD* anterior chamber depth, *ACV* anterior chamber volume.

*P value: The p-values after Bonferroni correction.

## Discussion

Using data from multiple centers, we revealed the correlations of CC and other anterior segment biometrics in young myopic adults. The findings are useful for better understanding of the interactions of anterior segment structures in myopia.

CC was higher in females than in males in the present study. This is consistent with many other previous studies^6,10,13^. In a population-based study, both the horizontal and vertical corneal refractive power was higher in women than in men^10^. In a study with healthy young university students (mean SE − 4.1 ± 2.7 D), although there was no sex difference in refractive errors, female participants had higher CC than male participants^6^. In highly myopic patients with the mean age of 31.2 ± 16.5 years, females were also shown to have higher CC than males^13^. In the present study, CC was higher in female participants than in male participants across all myopia groups, suggesting sex difference in corneal characteristics of myopia patients. One of the reasons for higher CC in females may be due to shorter axial length (AL) in women than men, and more corneal refractive power is needed to converge the light in female eyes^6,19^. Another reason is that higher CC in females is required for better emmetropization to prevent further myopic shift due to AL elongation^20^.

The correlations of CC and other anterior segment biometrics were also demonstrated in the present study. CC was negatively correlated to WTW after adjusting for age, sex and SE. This was consistent with previous studies conducted in 4–18 years old children^21^ and cataract patients^22^. A possible explanation of the negative correlation of CC and WTW is that CC is lower in taller subjects^21,23^, who have been shown to have larger WTW^24^. Another mechanism is that CC is decreased with AL elongation^10,13^, but the WTW is increased with longer AL^25^. We also demonstrated a negative correlation of CC and CCT, and similar results were also reported in previous studies with young subjects^12,26^. However, the correlation of CC and CCT may be positive in the elderly^27,28^. CC was also negatively correlated to CV-3 mm in the present study, but CC was not correlated to CV-5 mm, and was positively correlated to CV-7 mm, suggesting that the correlation of CC and CV may differ according to the area of interest.

CC was shown to be positively correlated to ACA and PCA. Only few studies have reported the correlation of CC and astigmatism. In the Shandong Children Eye Study, lower corneal refractive power was correlated to smaller cylindrical refractive error^21^. In another study with university students, lower corneal astigmatism was also observed in participants with lower corneal refractive power^29^. However, none of the previous studies has evaluated the correlation of CC and PCA. Our study showed that CC was correlated not only to ACA, but also to PCA, suggesting changes of corneal refractive power may affect both the anterior and posterior corneal toricity in myopia, and such changes are more prominent in some meridians.

CC was correlated to anterior and posterior corneal eccentricity and asphericity, including ACE, ACAP, PCE, and PCAP. Similar correlations were observed in a study with subjects from 6 to 83 years old and another study with the elderly^30,31^. According to these findings, when the cornea is steeper, it is also more prolate and eccentrical on the anterior surface but less prolate and eccentrical on posterior surface. It would be meaningful to investigate whether a steeper cornea is associated with more postoperative corneal eccentricity and asphericity, which are important parameters of visual quality after refractive surgery.

For biometrics of the anterior chamber, CC was positively correlated to ACD but negatively correlated to ACV. Since in the present study the CC reflected the steepness of the central 3 mm cornea, and the ACD showed the depth of the central anterior chamber, it was expected that a steeper central cornea was associated with a deeper central anterior chamber. Similar findings were also reported in a general population^10^. However, negative correlation of CC and ACD may be present in subjects of older age (40–80 years)^32^. Higher CC is also correlated to smaller WTW^10,21,29^, and smaller WTW is correlated to smaller ACV^33^. This may explain the negative correlation of CC and ACV in myopic patients. It is noteworthy that in patients with shallow anterior chambers, the correlation of CC and ACV may not exist^34^.

It is well known that CC is correlated to many other ocular biometrics in normal subjects. We revealed that CC is also correlated to other anterior segment biometrics in myopic adults. The correlations help us better understand the interactions of different anterior segment structures in the development of myopia. In myopic eyes, when the CC is steeper, WTW is smaller, CCT is thinner, corneal toricity, anterior corneal eccentricity and asphericity are higher, ACD is deeper, and ACV is smaller. The changes of these anterior segment structures may affect the development of myopia, or may be the consequences of AL elongation. Further studies are required to clarify the roles of these biometrics in myopia.

In conclusions, CC was correlated to most of the other anterior segment biometrics in young myopic adults, suggesting the interactions of different anterior segment structures in myopia. The sequential order of the changes of these structures in the development of myopia need to be further investigated.

## Data Availability

Data are available from the corresponding author upon reasonable request.
